# The role of microglia in the prion-like transmission of protein aggregates in neurodegeneration

**DOI:** 10.1093/braincomms/fcaf087

**Published:** 2025-02-25

**Authors:** Muhammet M Öztürk, Jakob Emgård, Juan García-Revilla, Rosalía Fernández-Calle, Yiyi Yang, Tomas Deierborg, Tomas T Roos

**Affiliations:** Experimental Neuroinflammation Laboratory, Department of Experimental Medical Science, Lund University, Lund 221 84, Sweden; Experimental Neuroinflammation Laboratory, Department of Experimental Medical Science, Lund University, Lund 221 84, Sweden; Experimental Neuroinflammation Laboratory, Department of Experimental Medical Science, Lund University, Lund 221 84, Sweden; Experimental Neuroinflammation Laboratory, Department of Experimental Medical Science, Lund University, Lund 221 84, Sweden; Experimental Neuroinflammation Laboratory, Department of Experimental Medical Science, Lund University, Lund 221 84, Sweden; Experimental Neuroinflammation Laboratory, Department of Experimental Medical Science, Lund University, Lund 221 84, Sweden; Experimental Neuroinflammation Laboratory, Department of Experimental Medical Science, Lund University, Lund 221 84, Sweden

**Keywords:** Microglia, prion-like, alpha-synuclein, amyloid-beta, tau

## Abstract

Numerous neurodegenerative diseases such as Alzheimer's disease, Parkinson's disease and amyotrophic lateral sclerosis share a neuropathological hallmark: aberrant protein aggregation in the CNS. Microglia, the brain's innate immune cells, also play a pivotal role in the pathogenesis of these disorders. Multiple studies indicate that these pathological aggregates can propagate throughout the brain in a prion-like manner. A protein/peptide that adopts a prion-like conformation can induce homologous proteins to misfold into a prion-like conformation through templated seeding, enabling cell-to-cell spread and accelerating protein aggregation throughout the brain. Two important questions in the prion-like paradigm are where the prion-like misfolding occurs and how the prion-like aggregates are spread throughout the CNS. Here, we review the role of microglia and associated inflammation in the prion-like spread of pathologically aggregated proteins/peptides in Alzheimer's disease, Parkinson's disease and amyotrophic lateral sclerosis. A growing body of evidence suggests that microglia can internalize prion-like proteins and transport them to neighbouring neurons and other glial cells. Microglia may also influence the potential seeding of proteins in neurons and induce inflammatory pathways in their microenvironment. This review aims to broaden the understanding of the role of microglia in the prion-like spread of protein aggregation.

## Teaser

Microglia, the immune cells of the brain, are important in neurodegenerative disorders. They may also help spread diseased proteins within the brain, prion-like proteins. In this review, Öztürk *et al*. summarize the current knowledge on the role of microglia in prion-like transmission of proteins in the most common neurodegenerative diseases.

## Graphical Abstract

**graphical abstract:**
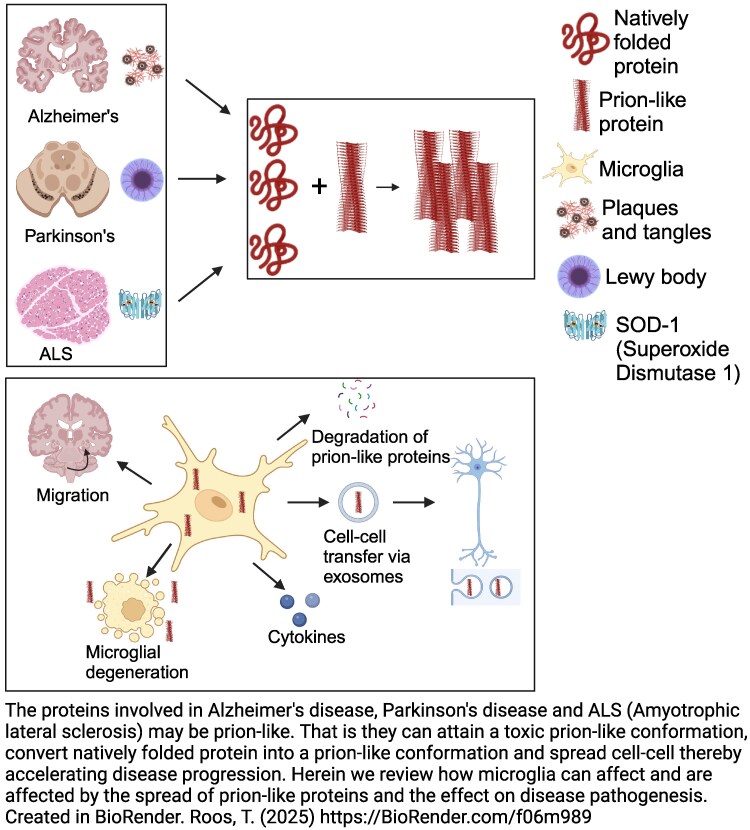

## Introduction

Toxic protein accumulation is a common neuropathological feature in many neurodegenerative diseases, including Alzheimer's disease, Parkinson's disease and amyotrophic lateral sclerosis.^1^ Each disorder is characterized by a particular misfolded protein or peptide: amyloid-beta (Aβ, a peptide) and tau in Alzheimer's disease,^2,3^ alpha-synuclein (α-syn) in Parkinson's disease^4^ and Cu/Zn superoxide dismutase 1 (SOD1), transactivating DNA-binding protein 43 (TDP43) or fused in sarcoma (FUS) in amyotrophic lateral sclerosis.^5-7^ Presumably, what distinguishes the clinical manifestations of each disorder is the misfolding of a specific protein at a specific origin and spread within the nervous system. In recent years, mounting evidence from animal and *in vitro* models has strengthened the prion-like hypothesis for the pathogenic spread of protein assemblies in Alzheimer's disease, Parkinson's disease and amyotrophic lateral sclerosis.^8-10^ Human case reports indicate the prion-like spread of α-syn in patients who received cerebral embryonic stem cell transplants.^11^ Similarly, recipients of cadaveric human growth hormone (hGH),^12^ dura mater grafts^13^ and those who underwent childhood neurosurgery^14^ showed signs of Aβ prion-like spreading in blood vessels (cerebral amyloid angiopathy). In early 2024, it was even published^15^ that some recipients of cadaveric hGH developed mid-life cognitive impairment with signs of Alzheimer's disease pathology. hGH recipients are closely monitored as prion-contaminated hGH has caused hundreds of cases of iatrogenic Creutzfeldt Jakob's disease, a true prion disorder. Eight hGH recipients were assessed for cognitive problems, and Creutzfeldt Jakob's disease was dismissed. Of these eight, five had early-onset dementia, and of those, two had biomarker changes consistent with Alzheimer's disease and one had neuropathological changes consistent with Alzheimer's disease; none had any known genetic form of Alzheimer's disease. Early-onset Alzheimer's disease is rare, so it is noteworthy that it seems more common among the recipients of hGH, though the number of cases is too small to state this with certainty. Furthermore, the recipients of hGH often have comorbidities and are therefore not directly comparable to the general population. Finally, even if we accept these cases as iatrogenic Alzheimer's disease, the transmission would be vastly less potent than that of prion protein. The prevalence of Creutzfeldt Jakob's disease is much lower than that of Alzheimer's disease, and yet, we have hundreds of cases of iatrogenic Creutzfeldt Jakob's disease and only a handful of possible cases of iatrogenic Alzheimer's disease.^15^ In summary, there is a little evidence for iatrogenic Alzheimer's disease or Parkinson's disease caused by the prion-like spread of pathological proteins/peptides and no evidence for either inter-individual or zoonotic spread as with the very rare, pure prion disorders.

Prion-like spread of a protein requires three fundamental features: (i) prion-like aggregate must convert natively folded proteins of the same type into the misfolded form thereby creating new prion-like aggregates; (ii) host cells must repeatedly generate peptide in question to remain ‘infected’ by aggregates, that is, you cannot induce prion-like spread into a host that does not express the protein in question; and (iii) toxic aggregates must spread/transmit between cells. The propagation of misfolded proteins from affected to unaffected regions may be a major cause of neuronal dysfunction and loss.

Most past studies have focused on the neuron–neuron spread of prion-like misfolded proteins.^16^ Nonetheless, multiple neurodegenerative disorders, including Alzheimer's disease, Parkinson's disease and amyotrophic lateral sclerosis, also contain insoluble protein deposits in astrocytes, oligodendrocytes and other glial cells.^17-20^ Microglia, the main phagocytic cells of the brain, may be of particular interest. Microglia phagocytose tau,^21^ Aβ,^22^ α-syn,^23^ TDP-43^24^ as well as whole neurons and synapses which may contain the proteins/peptides above.^25^ Activated microglia are also found in the vicinity of the insoluble protein aggregates triggering an inflammatory response.^26^ Furthermore, in contrast to other immune cells, microglia have exceptional longevity with a half-life of more than 15 months in mice^27^ and more than 4 years in humans, with some lasting more than two decades.^28^ Alzheimer's disease and Parkinson's disease have slow progressions; the neuropathological process starts years before overt clinical signs. In Alzheimer's disease, a pathological Aβ42/40 ratio can be detected in CSF more than a decade before the onset of clinical dementia.^29^ It is similar in synucleinopathies where pathological α-syn can be identified with the RT-QuiC assay, years before diagnosis.^30^ In the cases of suspected iatrogenic spread of misfolded α-syn and Aβ, it seemed to take several decades to develop any disease.^11-14^ The initial protein/peptide misfolding into a prion-like conformation also seems to be a slow process. Sporadic cases of Alzheimer's disease and Parkinson's disease are almost exclusively diseases of old age, and even in familial cases, clinical disease onset before middle age is rare. Microglia are also mobile,^31^ particularly in response to damage.^32^ Thus, microglia have a potential ability to develop and spread prion-like protein aggregates throughout the CNS due to their long lifespan and mobility. However, microglia are likely not the only glial cell that plays a role in prion-like propagation of proteins. Astrocytes are large cells, and one astrocyte can have contact with millions of synapses potentially facilitating cell-to-cell spread of proteins.^33^ They are also long-lived, but they lack the same mobility as microglia, even though they can migrate to injured tissue.^34^ Astrocytes also likely contribute to the propagation of prion-like proteins/peptides, particularly tau.^35^ Oligodendrocytes are also implicated. The synucleinopathy multiple system atrophy is characterized by α-syn inclusions in oligodendrocytes, and these inclusions are capable of prion-like seeding.^36^

In this review, we will discuss the role of microglia in the propagation of misfolded proteins in a prion-like manner and the role of microglia-derived inflammatory cytokines in the progression of prion-like spread. Many of the proteins (tau in particular) seem to be responsible for several different diseases, possibly related to their particular conformation/strain.^37^ We will, however, focus on the most common disorders protein by protein.

## Alzheimer's disease: microglia in the spread of tau and Aβ

Alzheimer's disease is the most common type of dementia. The neuropathological hallmarks are extracellular senile plaques, mainly comprised of the Aβ peptide, and aberrant intraneuronal neurofibrillary tangles (NFTs) mainly comprised of hyperphosphorylated tau protein. Besides neuronal death, synaptic loss and protein deposits, neuroinflammation is a key feature in Alzheimer's disease pathogenesis.^38^ Microglia sense Aβ aggregates and remove them through phagocytosis.^39^ However, these chronically Aβ-activated microglia secrete pro-inflammatory cytokines, impeding Aβ removal and triggering neuronal death.^40^ Thus, microglia hold both potentially beneficial and detrimental effects that have been described as a double-edged sword.^41,42^

Neurons have traditionally been the focus in studies of neurodegenerative disorders. Nonetheless, recent clinical findings show a significant role of microglia and neuroinflammation in neurodegenerative disorders, particularly Alzheimer's disease.^43^ For example, genome-wide association studies have uncovered neurodegeneration-related genetic risk variants (e.g. TREM2) that are widely or even exclusively present in microglia.^44-49^ In a healthy brain, microglia perform various pleiotropic functions, such as synaptic pruning, clearance of apoptotic neurons and neuronal development support.^50^ However, continuous microglial activation in the brain aggravates the progression of Aβ and tau pathology in Alzheimer's disease and triggers additional detrimental effects, such as neuronal loss.^51-53^ Activated microglia cluster around Aβ plaques^54^ and adapt a unique gene expression profile, namely disease-associated microglia.^41,55^ Furthermore, activated microglia can also associate with neurons containing intraneuronal Aβ^56^ and neurons with NFTs.^57^ Finally, one of the best correlates to cognitive decline in Alzheimer's disease is loss of synapses,^58^ and microglia and astrocytes play a large role in engulfing synapses.^59,60^ This may seem detrimental, but considering that neuronal hyperactivity is a key feature of Alzheimer's disease (until the recent arrival of Aβ antibodies, the only medications against Alzheimer's disease targeted neuronal hyperactivity), phagocytosis of synapses may, thus, also be beneficial.^61^ Pruning hyperactive synapses may be beneficial in the short term, while doing so microglia could also engulf prion-like tau and Aβ, potentially accelerating disease progression—double-edged sword indeed. It has been shown that tau oligomers in synapses may constitute an ‘eat me’ signal,^60^ raising the question of whether this oligomeric ‘eat me’ tau has prion-like activity.

## Microglia in the spread of tau

Tauopathies, diseases caused by intracellular tau aggregation, encompass multiple neurodegenerative disorders, and Alzheimer's disease is by far the most common. Curiously though, no tau mutations are associated with familial Alzheimer's disease. There is considerable evidence that tau is capable of prion-like spread, meaning that tau can misfold into a prion-like conformation that can convert native tau into a prion-like, pathological, conformation and spread between cells. Intracerebral injection of brain homogenate as well as preformed tau fibrils can accelerate tau misfolding in mice that express human tau. This prion-like spread can also be produced and easily quantified in a tau expressing cell line (HEK-293) based on fluorescence resonance energy transfer (FRET), a model that has been used in many studies.^62^

Tau can be secreted and taken up by neighbouring cells, including microglia.^21,63-66^ Activated microglia phagocytose secreted tau aggregates *in vivo* and *in vitro* to regain homeostasis,^57,67,68^ and tau aggregates are found in reactive microglia in patients with Alzheimer's disease.^69,70^ In addition, microglia engulf synapses and even whole neurons containing pathological tau.^57,71^ Interestingly, a recent study reported that microglia that phagocytose tau-containing live neurons became hypophagocytic while expelling tau aggregates capable of prion-like seeding.^72^ Another study showed that microglia from both human Alzheimer's disease cases and a tauopathy mouse model contained tau capable of *in vitro* tau seeding, that is, prion-like spread.^70^ Maphis *et al*.^73^ used the hTauCx3cr1^−/−^ mouse, which expresses human tau in combination with overactivated microglia, to show that microglial activation is associated with the spread of tau pathology in the synaptically connected regions of CA1 and subiculum in the hippocampus. Their results indicated that local activation of microglia preceded the spreading of phosphorylated pathological AT180^+^ and AT8^+^ tau suggesting a role of activated microglia in the neuron–neuron transmission of pathological tau. Post-mortem studies have reported that the propagation of misfolded and accumulated tau tangles in Alzheimer's disease follows a conventional anatomical spread in the brain known as the Braak stages.^74,75^ However, whether microglia follow the same anatomical network as tau in the Braak stages remains unclear. Several animal studies suggest microglial involvement in the spread of tau pathology.^76-80^ A recent study in patients with Alzheimer's disease used PET to examine microglial activation, Aβ deposition and tau propagation in Alzheimer's disease brains.^81^ The authors hypothesized that microglial activation drives tau propagation throughout the brain in a Braak-like pattern in Alzheimer's disease. They showed co-localization of activated microglia that propagated across Braak stages, suggesting a microglial contribution to the stereotypical spread of tau in Alzheimer's disease. Just looking at the PET data, it seems that activated microglia precede tau deposition. However, PET has limited resolution so whether the microglial activation preceded any form of local tau (or Aβ) pathology is unknown. But the results do imply that a brain with activated microglia for some other reason provides fertile ground for prion-like spread of tau. Furthermore, the authors used [^11^C]PB28 tracer to measure microglial activation. [^11^C]PB28 tracer is a well-established marker for neuroinflammation and an indirect marker of microglia but cannot distinguish between exact functional states of microglia. Thus, the authors show joint propagation of tau and microglia but do not conclusively demonstrate whether the microglia are mainly involved in protective clearance mechanisms or contributing to the prion-like spread of tau. However, their findings suggest that the damaging effects of microglia predominate.

Hopp *et al*.^70^ demonstrated that microglia are unable to eliminate the prion-like seeding capability of tau. Thus, microglia should be capable of spreading prion-like tau throughout the brain. It has been suggested that tau secretion by microglia follows an exosome-dependent pathway.^76,77^ Tau-carrying exosomes have been detected in CSF and blood samples from patients with Alzheimer's disease and frontotemporal dementia.^82,83^ To confirm that exosome release mediates tau propagation, Asai *et al*.^76^ inhibited microglial exosome synthesis in transgenic P301S mice (these mice express human tau associated with frontotemporal dementia with parkinsonism), which led to a severe decline of pathological tau in the brain. In addition, microglial secretion of aberrantly phosphorylated and aggregated tau into extracellular space in mice has been shown, further supporting the role of microglia in tau spreading via an exosomal release mechanism.^77^ Mutated human tau, when expressed in the entorhinal cortex of mice via adeno-associated virus injection, becomes detectable in the dentate gyrus 1 month after injection, suggesting that the tau spread via synaptic connections since the entorhinal cortex and dentate gyrus are extensively connected via the perforant pathway.^78^ However, by depleting microglia, Audrian *et al*.^78^ observed reduced human mutated tau in the dentate gyrus. These findings suggest that prion-like tau propagation is not solely dependent on neuronal synaptic connections or other glial cells, and they are consistent with evidence that microglia also seemed to facilitate the neuronal Braak progression of tau.

There are also studies that examine the spread of tau in the context of microglia depletion. Asai *et al*.^76^ pharmacologically depleted microglia by inhibiting the colony-stimulating factor 1 receptor (CSF1R) in a mutated tau mouse model (the mutation, P301L is associated with frontotemporal dementia) and noted a significant decrease in At8^+^ cells as a result. However, in the 5xFAD mouse that has mutations in APP and its processing proteins but no tau mutations, there microglial ablation led to increased tau pathology.^84^ Perhaps, microglia are beneficial in the context of Aβ pathology but less so in pure tauopathy or it could just be due to the vagaries of the particular model. It should also be mentioned that CSF1R inhibition may have other effects as the CSF1R is not exclusively expressed in microglia. Macrophages, monocytes and even some non-immune cells express it, though microglia are particularly dependent on it for survival. Other glial cells may also partly compensate for the loss of microglia; astrocytes, for example, may upregulate phagocytosis and cytokine production in the absence of microglia.^85,86^

Microglial inflammation can contribute to the propagation of tau. One of the most studied and potent inflammatory pathways is the NOD-like receptor family pyrin domain-containing 3 (NLRP3) inflammasome, a multiprotein signalling complex that regulates the processing and release of pro-inflammatory cytokines, including IL-1β and IL-18. The NLRP3 inflammasome has emerged as a crucial mediator of microglial-driven inflammation in Alzheimer's disease. A recent study found that prion-like tau seeds can activate NLRP3-ASC inflammasome. A complex in which apoptosis-associated speck-like protein containing a CARD (ASC) functions as an adaptor protein, essential for inflammasome assembly and activation. They also found that ASC deficiency in P301S tau transgenic mice significantly reduced exogenously seeded tau pathology following a frontal cortex injection of pre-aggregated tau seeds at 3 months of age. Additionally, chronic intracerebral administration of the NLRP3 inhibitor, MCC950, suppressed exogenously and non-exogenously seeded tau pathology.^87^ Beyond the NLRP3 inflammasome, NF-κB is also an important regulator of neuroinflammation.^88^ This signalling pathway can be activated in microglia by pattern recognition receptors, damage-associated molecular patterns, pro-inflammatory cytokines and, pertinent to this review, pathological protein aggregates. Recently, the effect of microglial NF-κB signalling on tau seeding and spreading has been examined.^79^ Strikingly, elevated activation of microglial NF-κB by tau increased tau seeding and spreading in young P301S tau transgenic mice.^79^ Furthermore, Xu *et al*.^80^ reported that deficiency of microglial autophagy in P301S mice accelerated neuronal tau propagation and pathology, and notably, microglial-specific depletion of Atg7, essential for autophagosome formation, also triggered a pro-inflammatory state in microglia, potentially exacerbating tau spreading.

**Figure 1:**
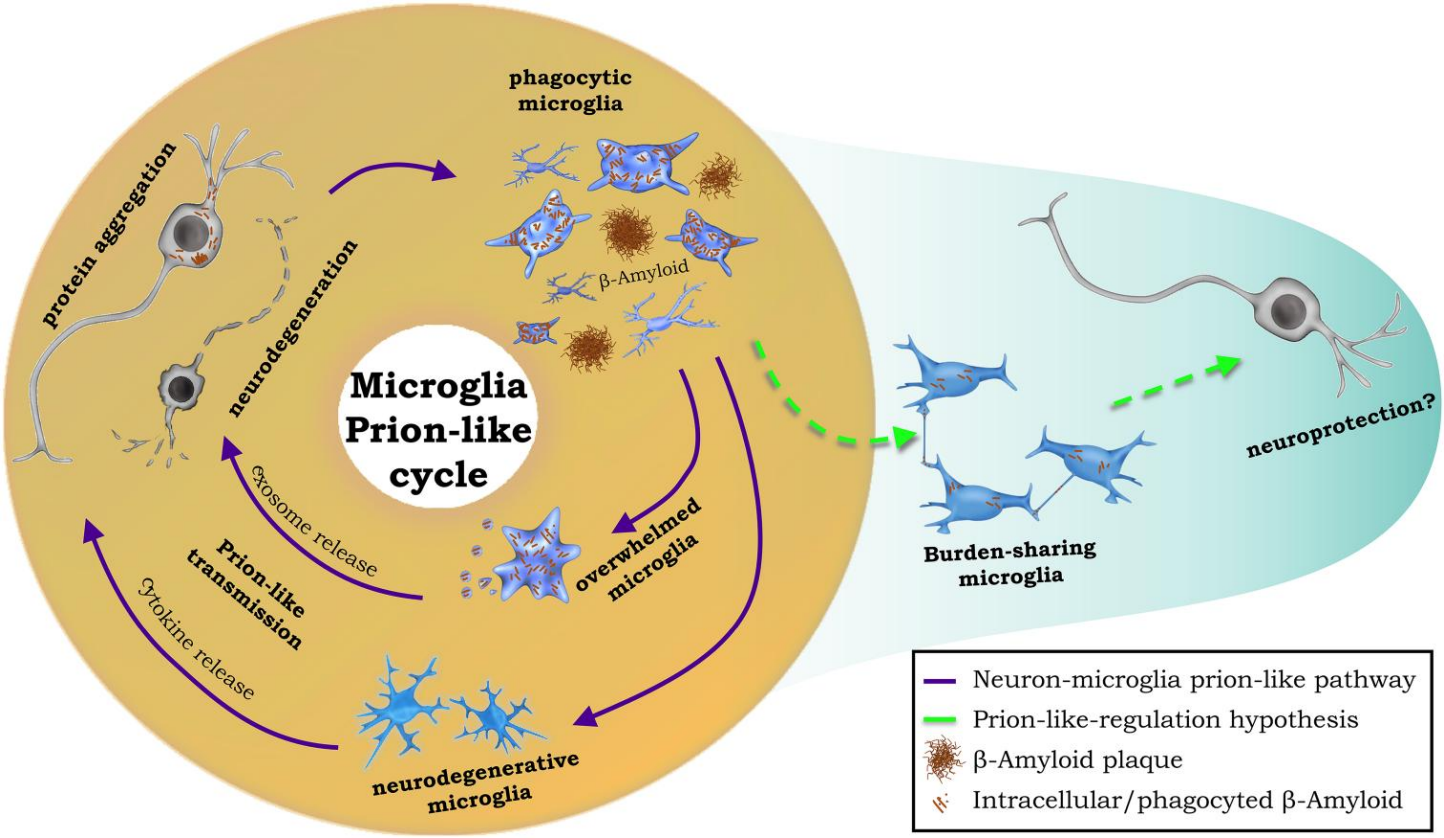
**Simplified proposed mechanisms for the microglia-related prion-like transmission of aggregated proteins.** Microglia take up prion-like protein aggregates from the extracellular environment or from a damaged neuron and become phagocytic which is beneficial. If there are too many aggregated proteins, then microglia cannot process it and the now overwhelmed microglia produce exosomes containing the prion-like protein or spread it between cells through some other mechanism. These overwhelmed microglia directly promote transmission of the prion-like aggregated protein to other microglia and neurons. In this environment (solid arrows), some microglia turn to a neurodegenerative phenotype characterized by the release of inflammatory mediators that contributes to neurodegeneration and prion-like transmission. Alternatively (dashed arrows), microglia can share the aggregated protein burden, e.g. through nanotubules, avoiding or delaying the surge of overwhelmed microglia. Whether microglia are primarily phagocytic and degradative (beneficial) or overwhelmed and releasing/spreading prion-like protein aggregates can explain the heterogeneous results of microglial depletion where it is sometimes helpful and sometimes harmful depending on the particular disease model and timing.

Mutated tau is not associated with Alzheimer's disease, yet most animal studies use mice with mutated tau, meaning that their findings may not be generalizable to Alzheimer's disease models, let alone to patients with Alzheimer's disease. Nevertheless, it is established that microglia take up both mutated and wild-type (WT) tau and contribute to its cell-to-cell spread. However, many studies do not explicitly test whether this propagating microglial tau also is capable of prion-like seeding, converting native tau to its prion-like form; but those that have investigated this^70,72^ found that tau in microglia and their exosomes are capable of prion-like seeding as assessed with the FRET biosensor cell line. Furthermore, microglia ameliorate but do not eliminate tau's seeding potential which would seem beneficial. However, microglia also seem to facilitate the spread of prion-like tau via exosomes, even human data support this claim.^81^ This dual role could also explain the somewhat contradictory effects of microglial depletion in different disease models. One can hypothesize a beneficial role of microglia when they help neurons to degrade prion-like tau aggregates; however, when microglia themselves become overloaded, they instead help the spread of prion-like tau. Whether the main activity of the microglia is degradative or propagative determines whether microglial depletion is beneficial. A simple illustration of this is presented in Fig. 1. A future challenge could be figuring out how to harness the beneficial effects of microglia, phagocytosing and degrading pathological tau aggregates, without facilitating the spread of prion-like tau.

## Microglia in the spread of Aβ

Mounting evidence supports prion-like transmission of Aβ aggregates contributing to disease progression in Alzheimer's disease. Already in 2000, it was shown that intracerebral injection of Alzheimer's disease brain homogenate accelerates plaque formation in an APP/Aβ expressing host^89^ which has been replicated many times.^90-94^ Furthermore, cortical and hippocampal tissues of WT and APP23 mice develop plaque pathology when transplanted into the transgenic APP23 mouse brain.^95^ However, the mechanisms of Aβ spread and cell-mediated transmission are not fully elucidated. Several studies report a role of microglia in Aβ seeding.^94,96,97^ d’Errico *et al*.^98^ showed a critical role of microglia by transplanting embryonic neuronal cells from WT mice into the neocortex of young 5xFAD^Cx3r1+/−^ transgenic mice. Four weeks later, they detected Aβ aggregates in the seeded WT grafts. Fourteen days after transplantation, a statistically significant amount of CX3CR1^+^ host microglia cells were in intimate contact with the WT grafts, possibly due to injury or the presence of graft itself, and a plenty of small Aβ particles were observed in or closely associated with the host microglia. Depletion of microglia abated, but did not eliminate, Aβ deposition inside the grafts. To rule out tissue injury, the authors co-cultured old transgenic brain slices with young WT brain slices. At day 14 *in vitro*, Aβ-bearing microglia that had migrated towards the WT tissue were detected, validating the hypothesis that microglia contribute to the spread of Aβ pathology into healthy brain tissue.^98^ In 2017, Venegas *et al*.^94^ showed ASC specks could accelerate the deposition of Aβ; the activation of the innate immune system in Alzheimer's disease involves the inflammasome-dependent formation of ASC specks in microglia. Furthermore, when Alzheimer's disease brain homogenate was injected into a 5xFAD mouse that did not express ASC or if the brain lysate was from a 5xFAD mouse that did not express ASC, prion-like seeding of Aβ was greatly attenuated. This indicates a significant role of microglia-derived ASC in the prion-like spread of Aβ in Alzheimer's disease, which has also been noted with tau.

Baik *et al*.^99^ reported that microglial cell death increased Aβ plaques *in vivo*. Long-term *in vivo* two-photon microscopy imaging confirmed that excessive uptake of Aβ leads to microglial cell death further elevating the amount of extracellular Aβ. As with tau, several studies have also examined the effect of microglia depletion on Aβ deposition, and as with tau, the results have been heterogeneous. Many studies have shown a decrease of plaques in 5xFAD mice via inhibition of CSF1R initiated at a relatively early age.^100-102^ However, in older 5xFAD mice, microglial depletion does not reduce plaque burden.^103^ The decrease of plaques in the 5xFAD is particularly notable as it was reported that microglial depletion in this model increased tau pathology. However, when 3xTG mice (treatment initiated at 15 months, 9 months after the first plaques) or APPPS1 mice (treatment initiated at 6 months, also several months after the first plaques) were treated with CSF1R inhibition, no change in plaque burden was observed.^104,105^ Nevertheless, other beneficial effects were noted. Other studies have genetically modified APPPS1 mice to have a suicide switch for myeloid cells, activated by either ganciclovir in 3-month-old mice^106^ or diphtheria toxin^107^ in 12-month-old mice. In the first case, microglial ablation caused no change in plaques, while in the second case, a small increase in plaques was observed. CSF1R inhibition thus seems to decrease plaques if initiated early, but not at later stages. Notably, the most dramatic effects have been observed in the highly aggressive 5xFAD mouse model. Genetic ablation did not reduce plaque burden in relatively young mice and even increased it in older mice. Notably, in the APPPS1 model, neither genetic modification nor CSF1R inhibition to ablate microglia reduced plaque burden. Considering the heterogeneity of the data, it would be useful with a more systematic investigation of the effect on both tau and Aβ burden after ablation of microglia with CSF1R inhibition with different mouse models and ages. Furthermore, as with tau, one can hypothesize a dual role of microglia. Microglial ablation during the early seeding phase of prion-like Aβ reduces plaque burden because cell-to-cell transmission is reduced without microglia. However, when Aβ pathology is already established, microglial ablation does not reduce plaque burden (Fig. 1), as the prion-like seeds are already dispersed. As with tau, most studies show that microglia influence the spread of Aβ but do not explicitly test its seeding capability. The closest is Venegas *et al*.,^95^ which showed that microglial ASC is essential for prion-like seeding of Aβ, suggesting that microglia are essential for the prion-like seeding of Aβ.^94^ While prion-like Aβ may also be intracellular,^108,109^ there is currently no *in vitro* model to assess seeding activity, as exists for tau. It would be worthwhile to directly test how microglial phagocytosis affects the seeding capability of Aβ. For example, microglia could be exposed to 5xFAD brain homogenate (containing Aβ capable of prion-like spread), and the microglial processed Aβ could then be intracerebrally injected to assess its prion-like activity.

## Parkinson's disease: microglia in the spread of α-syn

The aberrant cytoplasmic inclusions of α-syn in neuronal soma, known as Lewy bodies (LBs), and degeneration of dopaminergic neurons in the substantia nigra pars compacta are the major neuropathological hallmarks of Parkinson's disease.^110,111^ α-Syn is a 14-kDa protein, considered to be at the root of both familial and sporadic Parkinson's disease.^112,113^

The prion-like spread of α-syn pathology within the brain was proposed by Li *et al.*^11^ after the discovery of LBs in grafted embryonal neurons in patients with Parkinson's disease more than a decade after transplantation. Since cells should not autonomously develop LBs after 12 or 16 years, this observation indicated possible spread of the LB pathology. This evidence has been corroborated in other clinical cases, animal and cell culture models.^114-116^ The mechanism proposes that misfolded species act as templates (seeds) for naïve endogenous α-syn to adopt a fibrillar conformation and accumulate in neurons. Indeed, preformed fibrils (PFF) of α-syn have been shown to be an excellent model for reproducing α-syn pathology in cell culture and animal models, leading to a progressive accumulation of endogenous α-syn.^117,118^ Further affirming the idea of the prion-like α-syn spreading, it was demonstrated that a single intrastriatal inoculation of α-syn fibrils in mice leads to their transmission to anatomically connected regions and degeneration of the SN.^116^ Similarly, injection of fibrils in distant structures including the olfactory bulb^119^ or the gastrointestinal tract^120,121^ leads to accumulation of α-syn in the SN. These results align with Braak's hypothesis of Parkinson's disease, proposing that α-syn pathological misfolding can occur in any part of the nervous system, including the periphery (which would not be microglia dependent as they exist solely in the CNS), and would later propagate into the CNS particularly affecting the SN.^122,123^

Studies of prion-like spreading of α-syn have focused on neurons.^115,124-128^ However, increasing evidence suggests a prominent role for microglia in this process. Patients with Parkinson's disease exhibit chronic CNS inflammation, primarily mediated by microglia^129^ which activate in response to distinct forms of α-syn.^130^ Several studies have suggested that microglial activation can modify the prion-like spreading of α-syn. Recently, two independent studies pointed to Toll-like receptor-2 (TLR2)^131,132^ as a major microglia receptor responsible for α-syn spreading, while TLR4 may drive α-syn clearance.^133^ An interesting study by George *et al*.^134^ investigated the effect of microglia depletion, with CSF1R inhibition, and microglia activation in α-syn spreading into transplanted neurons in a mouse host that over-expressed α-syn. Surprisingly, both microglial depletion and lipopolysaccharide (LPS)-induced microglial activation increased α-syn transmission to grafted neurons. This indicates that microglia in untreated mice inhibit α-syn spread, as microglial depletion led to increased α-syn transfer. But LPS-activated microglia may promote α-syn spread rather than containing it. However, they did not directly compare LPS treatment with microglial depletion, so whether activated microglia are worse than depleted microglia is unknown. Several mechanisms can be involved in the transmission of misfolded and aggregated α-syn such as direct penetration of ‘naked’ α-syn, tunnelling nanotubes, glymphatic flow and exosomes.^128,135-138^ Extracellular vesicle trafficking including exosomes has been demonstrated to be relevant in microglia activity^139^ and has been suggested to change in response to α-syn. For instance, murine-derived microglial cell line (BV2) cells exposed to α-syn showed an increase in exosome release rich in tumour necrosis factor (TNF)-α which triggered neuronal apoptosis.^140^ Recent evidence reported that microglia actively contribute to the prion-like spread of α-syn through exosome release.^141^ In this study, the authors treated primary microglia with α-syn PFF, collected the exosomes and injected them into the mice brains. Injected mice elicited motor deficits, neurodegeneration and α-syn accumulation in the nigrostriatal pathway. Indeed, when microglia also were treated with pro-inflammatory LPS, α-syn accumulation increased, suggesting that the microglial phenotype affects α-syn transmission and aggregation. Intriguingly, they also demonstrated that the depletion of microglia in mice restrained the spread of α-syn after the inoculation of PFF, contrary to what was shown by George *et al*.^134^ These contrasting results may stem from different models: George *et al*. used neuronal α-syn overexpression, while Guo *et al*.^142^ used PFF injections with higher pathological α-syn loads, potentially leading to phagocytic versus overloaded microglia, respectively (see Fig. 1). Furthermore, Guo *et al*.^141^ also showed microglia/macrophage CD11b + exosomes containing α-syn oligomers in CSF from patients with Parkinson's disease that were capable of seeding α-syn aggregation in neurons. Recently, a two-photon microscopy approach was used to demonstrate that microglia can transfer misfolded α-syn from overloaded microglia to neighbouring acceptor microglia to ‘share’ α-syn burden *in vivo*. In that study, Scheiblich *et al*.^142^ suggested tunnelling nanotubules as the mechanism for transferring misfolded α-syn and organelles. Whether microglial cells also could transport α-syn into neurons through tunnelling nanotubules was not addressed. Although the study aimed to disclose unknown mechanisms underneath the microglial clearance of α-syn, the results suggest that the physical interaction between microglia may allow them to decelerate the prion-like spread of α-syn during the early stages of Parkinson's disease pathology.

Numerous findings, including from our lab, have reported that extracellular α-syn prompts inflammatory responses in glial cells, including microglia.^130,143-145^ We recently found that microglial protein like galectin-3 can shape toxic α-syn and alter its aggregation.^146^ It has also been demonstrated that specific α-syn overexpression in microglia triggered dopaminergic neurodegeneration through exacerbated inflammatory response.^147^ In the previously mentioned study by Guo *et al*.,^141^ they also addressed whether some pro-inflammatory cytokines could impact α-syn spread by microglial exosomes. *In vitro* neurons treated with microglial exosomes showed increased α-syn spreading when cytokines (TNF-α, ILβ and IL-6) were present, indicating that the internalization of microglial exosomes was increased by inflammation, causing increased α-syn spread. TNF-α also promoted α-syn secretion and cell-to-cell spread through senescence-associated lysosomal exocytosis.^148^ As with tau spread in Alzheimer's disease, recent evidence indicates a role of the NLRP3 inflammasome in the spread of α-syn in Parkinson's disease. Microglial NLRP3 activation may promote the release of exosomes,^149^ which can facilitate the spread of α-syn aggregates. It has also been shown that the inhibition of the NLRP3 inflammasome suppresses fibrillar α-syn-induced inflammasome activation in mouse microglia and prevents extracellular release of ASC. Moreover, oral administration of the NLRP3 inhibitor (MCC950) in Parkinson's disease models inhibited inflammasome activation, leading to improvements in motor function, protection against nigrostriatal dopaminergic neuron loss and reduced accumulation of α-syn aggregates.^150^ Another report also suggests that microglial inflammation may enhance prion-like spread of α-syn in the brain.^151^ Intracerebral injection of a variant of α-syn less capable of fibril formation in mice revealed a greater inflammatory response than WT α-syn. Interestingly, this variant also promoted more spreading of aggregates compared with the WT variant. When an anti-inflammatory agent was administered orally, it was shown that the spreading of α-syn aggregates, inflammation and behavioural deficits were improved. They also demonstrated increased cell-to-cell transmission of α-syn upon treatment of cells with inflammatory cytokines. It has also been proposed that targeting the sustained inflammation in the gastrointestinal and olfactory systems, which are thought to be initial spreading sites for α-syn,^152^ may reduce the chance of developing Parkinson's disease.^153^

In conclusion, microglia seem to spread prion-like α-syn aggregates primarily via exosomes, a process exacerbated by inflammation. As with tau and Aβ, microglia can play both positive and negative roles and there is some evidence that the negative effects are greatest when the microglia become overloaded, contrasted by George *et al*.^134^ and Guo *et al*.^141^

## Amyotrophic lateral sclerosis: microglia in the spread of SOD1 and TDP-43

Amyotrophic lateral sclerosis is a neurodegenerative disorder characterized by the progressive degeneration of upper and lower motor neurons (MNs). This manifests as a gradual loss of muscle function, leading to paralysis and eventually death due to respiratory failure. Amyotrophic lateral sclerosis is typically classified as either familial amyotrophic lateral sclerosis or sporadic amyotrophic lateral sclerosis, comprising ∼10 and 90% of all cases, respectively.^134,141,154^ Both familial amyotrophic lateral sclerosis and sporadic amyotrophic lateral sclerosis share histopathological features including neuronal and glial cytoplasmic inclusions mainly composed of ubiquitin-labeled aggregated proteins, and some of the most frequently observed are SOD1, TDP-43 and FUS.^7,155,156^ The symptoms at the onset of amyotrophic lateral sclerosis are typically focal and unilateral and manifest at seemingly arbitrary locations. As the disease progresses, symptoms tend to spread gradually from one muscle group to the next, appearing to reflect a segmental spread of degeneration in MNs throughout the spinal cord and brain.^157-159^ This neuroanatomical spread of pathology has been observed to follow two distinct patterns—first, propagation through the neural network via axonal and synaptic transmission and second, propagation through the extracellular matrix to neighbouring areas.^160^ The mechanisms underlying this spatiotemporal propagation of pathology in amyotrophic lateral sclerosis remain unclear, but accumulating evidence both *in vivo* and *in vitro* suggests that the pathological proteins in amyotrophic lateral sclerosis can spread intercellularly to seed the formation of new aggregates.^9,161-164^ Several mechanisms for cell-to-cell transmission of misfolded proteins have been proposed, such as transmission via exosomes, synapses, receptor-mediated endocytosis or micropinocytosis of proteins from dead cells.^165^ So far, most studies investigating this phenomenon have pointed towards neurons as its main driver. In addition to this, recent publications have also suggested that astrocytes directly contribute to the prion-like protein propagation in amyotrophic lateral sclerosis ^166,167^ However, microglia's role in spreading pathological aggregates in amyotrophic lateral sclerosis remains largely unexplored.

How microglia interact with, internalize and metabolize the pathological proteins and their aggregates in amyotrophic lateral sclerosis has not been investigated to the same extent as in, for example, Alzheimer's disease or Parkinson's disease. Most amyotrophic lateral sclerosis microglia studies have focused on other aspects, particularly their role in neuroinflammation and neurotoxicity.^168-170^ On the other hand, as in Alzheimer's disease and Parkinson's disease, there is a substantial body of evidence demonstrating that microglia may act in a neuroprotective fashion, highlighting their possibly heterogeneous role in amyotrophic lateral sclerosis.^171,172^ Microglial activation can be traced all the way back to the earliest stages of amyotrophic lateral sclerosis pathogenesis, but it has been widely proposed that microglia are more important during later stages of the disease, fitting with the idea that microglia might contribute more to the spread of pathology rather than its initiation.^173^ Evidence for microglial involvement in amyotrophic lateral sclerosis seeded propagation primarily comes from studies of SOD1, an amyotrophic lateral sclerosis-associated protein, an antioxidant enzyme that when mutated accounts for ∼20% of familial amyotrophic lateral sclerosis cases.^5^ Mutated and misfolded SOD1 can propagate intercellularly as well as confer its misfolded conformation onto natively folded WT proteins *in vivo*, qualifying as a possible prion-like protein.^174-176^ The specific role of microglia in this phenomenon has not been extensively described, but a few studies have investigated the role of mutant SOD1 in exosomes derived from or internalized by microglia. Pinto *et al*.^177^ investigated the effects of incubating exosomes isolated from SOD1-mutated (G93A) NSC-34 MN-like cells with N9 microglia. The exosomes were internalized by the microglia, triggering a phenotype exhibiting pro-inflammatory, M1-like characteristics and a 50% reduction of phagocytic ability. However, the authors did not investigate how transmitted SOD1 affected microglia. Another study by Massenzio *et al*.^178^ investigated exosomes using rat primary microglia expressing familial SOD1 mutations, revealed that mutated SOD1 accumulated intracellularly and could be detected in exosomes, albeit to a lesser degree than native SOD1 in WT microglia.^178^ Co-cultures with WT primary neurons further revealed that SOD1-mutated microglia alone were able to induce neurotoxicity, which was hypothesized to be in part due to increased microglial activation and a neuroinflammatory response. However, the study did not investigate whether co-cultured neurons internalized mutant SOD1 from microglial exosomes or whether this contributed to neurotoxicity, but it would be an important mechanism to examine in future studies. Vaz *et al*.^179^ investigated N9 murine microglial cells overexpressing mutant SOD1 (G93A) and also showed an enrichment of SOD1 in the microglia-derived exosomes. However, similar to the study by Massenzio *et al*.,^178^ the authors did not investigate whether the exosomal SOD1 could also be transmitted intercellularly. In contrast to these findings, one study analysed extracellular vesicles harvested from SOD1-mutated murine neural tissue, demonstrating that the extracted extracellular vesicles, containing abundant misfolded and aggregated SOD1, expressed only astrocytic and synaptic markers, but not microglial ones,^180^ suggesting that extracellular vesicles carrying misfolded SOD1 mainly come from other cell types than microglia.

Current knowledge about microglia's role in prion-like propagation of misfolded SOD1 in amyotrophic lateral sclerosis remains limited. There are only a few *in vitro* studies indicating that mutated SOD1 might be present in microglia-derived exosomes, but no evidence showing that the exosomal SOD1 can be propagated between cells. Furthermore, the finding that exosomes from microglia can carry misfolded SOD1 was not corroborated in the *in vivo* study by Silverman *et al*.^180^ Thus, given the scant and conflicting data on this topic, further studies investigating whether misfolded SOD1 is present in microglial exosomes and whether it can also be transmitted between cells will be important to understand the role of microglia in the propagation of SOD1 in amyotrophic lateral sclerosis.

In addition to the studies above investigating the direct role of microglia in the spread of pathological SOD1, there are a few studies suggesting that microglia might play a more indirect role in the spread of TDP-43, an RNA-binding protein that requires mislocalization from the nucleus to the cytoplasm to form aggregates.^181^ Several studies have previously shown that misfolded TDP-43 can propagate from cell to cell in a prion-like manner in amyotrophic lateral sclerosis,^9,163,182^ but few have mentioned the role of microglia in this regard. One study used real-time visualization to examine TDP-43 nucleo-cytoplasmic transport in degenerating zebrafish spinal cord neurons.^24^ The authors found that surrounding microglia readily phagocytosed the degenerating neurons and then observed rapid intracellular degradation of the engulfed TDP-43. They then suppressed microglial phagocytosis through PU.1-inhibition resulting in axonal and extracellular spreading of TDP-43. Given previously mentioned findings suggesting that amyotrophic lateral sclerosis -associated protein aggregates could propagate through axons and synapses, as well as through direct uptake from the extracellular environment,^165^ the results of this study suggest that functional phagocytosing microglia are of importance in the prevention of pathological TDP-43 spread. One could then speculate that targeting excessive microglial phagocytosis might inadvertently affect functional phagocytic microglia, potentially exacerbating TDP-43's prion-like spread. Another group studied how LPS-induced inflammation affected TDP-43 pathology in primary microglia from TDP-43-mutant mice and found increased mislocalization of TDP-43 from the nucleus to the cytoplasm as well as exacerbated TDP-43 aggregation.^183^ These results suggest activated microglia may promote TDP-43 seeding and prion-like spread by triggering increased TDP-43 aggregation through inflammatory responses. This is similar to the results of George *et al*.^134^ where LPS activation of microglia exacerbated the spread of α-syn. The role of microglial exosomes in TDP-43 spread remains unknown. A study by Iguchi *et al*.^184^ examined intercellular TDP-43 transmission via exosomes in primary cultures of neurons, astrocytes and microglia. They were able to detect TDP-43 in exosomes from neurons, but not from astrocytes or microglia.

In conclusion, evidence supporting a microglial contribution to prion-like propagation of toxic proteins in amyotrophic lateral sclerosis is limited, resting on data from few studies. Further studies should the role of exosomes and other protein transmission mechanisms to determine microglia's contribution to pathological protein spread in amyotrophic lateral sclerosis. Though, the absence of evidence and in some cases evidence of absence (no microglial exosomes with TDP-43 for example) points to a less prominent role of microglia in the prion-like spread of toxic proteins in amyotrophic lateral sclerosis than in Alzheimer's disease and Parkinson's disease. Considering that amyotrophic lateral sclerosis, unlike microglia, is not limited to the CNS, this is perhaps not so surprising.

## Conclusion

There is substantial evidence that microglia play an important role in transmitting tau, Aβ and α-syn, both directly through uptake and release and indirectly through their phagocytic and degradative activities that modulate the amount and possibly conformation of the proteins (see Table 1 for a summary). If one already accepts Aβ, α-syn and tau as prion-like proteins, then their microglia-dependent transmission demonstrates microglia's important role in spreading prion-like proteins. However, there are also studies, albeit fewer, more directly confirming that microglia can take up prion-like α-syn and tau and transmit them via exosomes and seed new aggregates, fulfilling criteria for prion-like spread.^70,72,141^ In the case of Aβ, microglia were also essential for prion-like seeding *in vivo,* as it was greatly attenuated without microglial ASC.^94^ However, it was not directly showed that the microglia took up and spread prion-like Aβ. The role of microglia in the prion-like spread of the amyotrophic lateral sclerosis-associated proteins is also not well studied, but it is reasonable to assume that it is smaller than for tau, Aβ and α-syn. Astrocytes seem to play a larger role here. Few studies examine how microglia influence the seeding capacity of prion-like proteins; the focus is mostly on the propagation. Tau is the most well-studied protein, as it has a convenient *in vitro* model to quantify seeding. Studies have shown that microglia can ameliorate, but not eliminate, tau seeding capacity.^70^ It would be interesting to study how microglial processing affects the seeding capacity of the other prion-like protein aggregates. Finally, microglia themselves do not express either Aβ, tau or α-syn to any appreciable degree. It is therefore more parsimonious to hypothesize that the first misfolding to a prion-like state occurs in a neuron for the intracellular proteins tau and α-syn. Aβ also exists intracellularly, and the first seed may originate there, though it is more controversial.^93,108,185^ However, in a brain with well-functioning phagocytic microglia, the first neuronally produced prion-like seed may be simply exocytosed and then phagocytosed and degraded by microglia. In contrast, in a brain with already overloaded microglia, the seed may instead spread throughout the brain.

**Table 1:** Short summary of potential microglial prion-like protein transmission mechanisms

Disease	Pathological protein	Microglial transmission mechanisms
Alzheimer's disease	Tau	**Phagocytosis and release:** Activated microglia engulf extracellular tau aggregates, but incomplete degradation leads to expelling of tau aggregates capable of prion-like seeding and spread to neighbouring cells.^72,73,81^ **Exosome-mediated spread:** Microglia-derived exosomes containing tau aggregates facilitate intercellular spread and seeding of tau pathology.^76,77,82,83^ **Inflammation-exacerbated spread:** Microglial activation enhances prion-like tau seeding and spreading.^79,80^ **Microglial depletion effects:** Conflicting results show microglial depletion can enhance or reduce tau seeding and spreading, depending on the model and context.^76,78,84^
Alzheimer's disease	Aβ	**Phagocytosis and migration:** Activated microglia internalize Aβ aggregates and can migrate to healthy tissue, contributing to the prion-like spread of Aβ.^98^ **ASC-dependent seeding:** Microglial ASC specks facilitate the prion-like Aβ formation, seeding and deposition of Aβ aggregates.^94^ **Microglial depletion effects:** Conflicting results show microglial depletion can enhance, reduce or not affect Aβ seeding and spreading. This likely depends on the animal model, age and method of reducing microglia.^100-107^
Parkinson's disease	Alpha-synuclein (α-syn)	**Exosome-mediated spread:** Microglia exposed to preformed fibrils of α-syn release exosomes containing α-syn oligomers capable spreading and seeding α-syn aggregates.^141^ **Inflammation-exacerbated spread:** Microglial pro-inflammatory cytokines (e.g. TNF-α, IL-6 and IL-1β) enhance α-syn spread in combination with microglial exosomes *in vitro* or even without exosomes.^141,148,149,151^ **Microglial overload and burden sharing:** Overloaded microglia transfer misfolded α-syn to neighbouring microglia via tunnelling nanotubes, redistributing the burden but potentially facilitating the prion-like spread of α-syn.^142^ **Microglial depletion effects:** Conflicting results show microglial depletion can either enhance or reduce α-syn spread, depending on the model and context.^134,141^
Amyotrophic lateral sclerosis	SOD-1	**Exosome-mediated role?:** Mutated SOD1 has been detected in microglia-derived exosomes *in vitro*,^178,179^ but evidence for intercellular propagation of pathogenic SOD1 via these exosomes remains unconfirmed. **Limited evidence:** *In vivo* studies suggest that extracellular vesicles carrying SOD1 primarily originate from other cell types, such as astrocytes or neurons.^167,180^
Amyotrophic lateral sclerosis	TDP-43	**Phagocytosis of degenerating neurons?:** Functional microglia may prevent TDP-43 spread indirectly by phagocytosing degenerating neurons containing TDP-43,^24^ but there is no direct evidence that microglial phagocytosis actively blocks extracellular or axonal spreading of TDP-43 aggregates. **Inflammation-induced aggregation?:** LPS-induced microglial inflammation increases TDP-43 mislocalization from the nucleus to the cytoplasm and exacerbates its aggregation,^183^ which might potentially enhance prion-like seeding and spreading of TDP-43 aggregates.

Many studies emphasize that microglia can play positive and negative roles, acting as a double-edged sword. We can only agree with this assessment. The varied results on protein aggregation under microglial depletion demonstrate this well. The effect of microglial ablation appears to depend on disease stage: during initial seeding, ablation reduces protein aggregation, while showing smaller or even opposite effect at later stages. The phenotype of microglia also seems important; LPS-induced activation, for example, was detrimental. It would be useful for studies investigating prion-like spread to elucidate the role of microglia using different types of inflammagens such as poly-IC (TLR3 agonist), TNF-α and cytokines (e.g. IL4 and IL10). We hypothesize that microglia are beneficial when they primarily degrade prion-like aggregates in place. However, when overwhelmed, they secrete and transmit these aggregates throughout the brain. Promoting phagocytic microglia that efficiently degrade protein aggregates and avoiding overloaded microglia that spread them may be important for any treatment attempting to modulate neuroinflammation in neurodegeneration.

## Data Availability

Data sharing is not applicable to this article as no new data were created or analysed in this study.
